# No “carry-over” effects of tracking devices on return rate and parameters determining reproductive success in once and repeatedly tagged common swifts (*Apus apus*), a long-distance migratory bird

**DOI:** 10.1186/s40462-022-00357-y

**Published:** 2022-12-08

**Authors:** Arndt H. J. Wellbrock, Klaudia Witte

**Affiliations:** 1 Research Group of Ecology and Behavioural Biology, Institute of Biology, University of Siegen, Adolf-Reichwein-Straße 2, 57076 Siegen, Germany; 2 Institute of Avian Research “Vogelwarte Helgoland”, Wilhelmshaven, Germany

**Keywords:** Long-term study, Breeding parameter, Individual consistency, Geolocation, GPS, Apparent survival

## Abstract

**Background:**

To understand life-history strategies in migratory bird species, we should focus on migration behaviour and possible carry-over effects on both population and individual level. Tracking devices are useful tools to directly investigate migration behaviour. With increased use of tracking devices, questions arise towards animal welfare and possible negative effects of logger on birds. Several studies were conducted to address this question in birds that were tagged and tracked for one complete non-breeding season including migration but with mixed results. To detect individual-based decisions regarding migration strategy, we need to track the same individuals several times. So far, there are no studies investigating effects of repeatedly tagging on reproduction and life-history traits in individual migratory birds, especially in small birds.

**Methods:**

We used long-term data of 85 tagged common swifts (*Apus apus*), a long-distance migratory bird, of a breeding colony in Germany to test whether carrying a geolocator or GPS logger once or repeatedly during non-breeding season affected return rate, apparent survival, and parameters determining reproductive success. Additionally, we checked for individual differences in arrival date and breeding parameters when the same individuals were tagged and when they were not tagged in different years. Further, we calculated the individual repeatability in arrival at the breeding colony and date of egg laying in repeatedly tagged swifts.

**Results:**

Once and repeatedly tagged birds returned to the colony at a similar rate as non-logger birds and arrived earlier than non-logger birds. We found no effect of logger-type on return rate in logger birds. We detected no differences in apparent survival, time lag to clutch initiation, date of clutch initiation, clutch size, number of chicks and fledglings between logger and non-logger birds. We found neither an effect of loggers nor of logger-types on the arrival date and breeding parameter on individual-level. Arrival date was highly repeatable and date of clutch initiation was moderately repeatable within repeatedly tagged individuals.

## Background

Long-distance migratory birds often migrate between continents and live in different worlds within each year. Living in different parts of the world means facing different challenges. These can be very variable: from climatic factors to the availability of food [1, 2]. Some long-distance migratory birds spent even most of their lifetime at their wintering grounds and only a short period of time at their northern breeding grounds [3, 4]. To understand life-history strategies in migratory bird species, it is not sufficient to monitor the breeding season. Instead, we should also focus on non-breeding periods to learn more about important parameters influencing migration behaviour and possible carry-over effects on life-history traits, i.e. processes in the previous season that affected the breeding success of an individual in the following season and vice versa [5].

Thanks to tracking devices such as light level-geolocators (hereafter “geolocators”), GPS loggers or radio transmitter, we gained knowledge about migration behaviour [4], like timing of migration, migration tracks, locations of overwintering areas and the time they spend in each area [i.e. 6–8]. All this information contributes to a better understanding of life-history strategies in long-distance migratory bird species [9, 10]. In recent years, tracking devices are getting smaller and lighter so that even small bird species (less than 100 g) can be tracked [11–14], and the number of biologging studies on birds is constantly increasing [15–18]. So far, many studies investigated ecological carry-over effects in migratory species, i.e. factors, circumstances and/or constraints an individual faces in one season, i.e. during the overwintering season, that may affect the performance of that individual during the next breeding season and vice versa [e.g. 5, 19]. With the growing number of biologging studies, questions arise regarding animal welfare and possible negative effects on reproductive success and life-history traits due to carrying a tracking device, i.e. possible “carry-over” effects in these bird species. These additional effects may even mask the actual effects of interest. Some studies have shown that there are no effects of tagging on survival or reproduction success [20–24]. Important to note is that one study found injuries on some of the tagged birds but no negative relationship between being tagged and return rate, hatching and fledgling success were found [21]. Other studies detected negative effects on return rate and survival [25, 26] and breeding such as delay in clutch initiation, reduced breeding success and reduced parental care [27–30]. A meta-analysis of 74 published and 48 unpublished paper could detect only a weak effect on apparent survival [31].

To gain more insight into life-history strategies, we need more details on decisions on individual level within a species. For this, it is essential to track the same individuals several times, which will provide information about the consistency of individual migration behaviour [e.g. 13, 32–36]. The number of studies using repeated tracking in bird species is expected to increase [14, 24, 34, 37]. So far, there is, however, no data at all on possible effects of repeated tagging on migratory birds regarding traits influencing reproductive success. Thus, we need more studies investigating effects of tagging once and even more important long-term studies focussing on effects of repeated tagging on migratory birds. By comparing return rates, apparent survival, and breeding parameters in logger birds which were tagged once or repeatedly tagged with non-logger birds, we can assess whether this technique affects important parameters influencing reproduction. Here, we investigated possible “carry-over” effects, i.e. effects due to carrying a geolocator and/or a GPS-logger once and repeatedly in common swifts *Apus apus* using our long-term data covering 2012–2020.

The common swift is a small (about 40 g), highly aerial long-distance migratory bird species with breeding sites throughout Europe. The breeding season lasts from April until the end of August / beginning of September, with a shorter breeding season in the south than in the north of Europe. In general, a breeding pair has one clutch per breeding season with up to three chicks [38, 39]. Swifts are strictly insectivore and catch the food exclusively in the air. Outside the breeding season, common swifts spend almost 10 months continuously on the wing and overwinter in sub-Saharan Africa as far as south Africa [13, 40–42].

We studied swifts breeding at a location in Germany and investigated whether return rate to the breeding colony, apparent survival, and breeding parameters like arrival date at the breeding colony, time period to clutch initiation after arrival, date of first egg, clutch size, number of chicks, and number of fledglings are affected by tagging once and/or repeated tagging, and type of logger (with or without a light stalk) of one of the parent birds in the previous year. We compared these parameters in logger birds which carried a logger once or up to five times in different years about 9–10 months before returning to the breeding site with those in non-logger birds returning to the same breeding site in the same year. We studied a logger effect on individual level in two ways: (1) We compared arrival date and breeding parameters within same individuals when they were tagged and when they were not tagged. (2) In repeatedly tagged logger birds, we measured repeatability in arrival date and date of clutch initiation over several years. If loggers have an effect on apparent survival, we would expect a lower return rate in once tagged and/or repeatedly tagged logger birds than in non-logger birds. Additionally, in the case of a negative effect of tagging, we would expect once and/or repeatedly tagged logger birds to arrive later than non-logger birds, and an individual bird to arrive later when it is tagged than when it is not tagged. We assume that delayed arrival will have an impact on breeding parameters.

## Methods

### Study side and employment of loggers

#### Common swift breeding colony

The breeding site is located in a walk-in concrete bridge near the city of Olpe, North Rhine-Westphalia, Germany (51°02ʹ28ʹʹN, 7°49ʹ36ʹʹE) [43]. According to the geographical location, breeding season lasts from the end of April to the beginning of August. Since 2007, we ring adult and juvenile birds (aluminium ring), and equipped them with a RFID transponder (trovan ID-100A (1.4), trovan™, Frechen, Germany) for individual identification. Birds were automatically read by loop antennas located around the nest or around the entrance hole near by the nest. The number of breeding pairs has increased steadily from 38 breeding pairs in 2007 to 62 breeding pairs in 2020.

#### Tracking devices

To track the swifts throughout their non-breeding season, we equipped 76 adult swifts with archival light-level geolocators from Biotrack Ltd (Wareham, UK) or the Swiss Ornithological Institute (Sempach, Switzerland) and nine adult swifts with GPS-logger from PathTrack Ltd (Otley, UK) with a full body harness [13, 41] between 2012 and 2019 (in total N = 85, including 16 repeatedly tagged birds, Table 1). Geolocators/GPS-Loggers plus full body harnesses constituted 1.4–4.1% of the individuals’ body mass with average body mass of swifts of 43.8 ± 3.73 g (mean ± SD).

**Table 1:** Overview of logger types (company, weight and presence of light stalk) and mean weight of logger birds and non-logger birds in the years 2012–2019

Year	Logger type	Company	Logger weight including body harness [g]	Light stalk	Mean weight of logger birds [g]	Mean weight of non-logger birds [g]
2012	MK5540	Lotek (former Biotrack Ltd), Wareham, UK	0.68	No	40.1 ± 2.7	44.4 ± 2.3
2013	ML6590	Lotek (former Biotrack Ltd), Wareham, UK	0.79	No	45.9 ± 2.9	40.8 ± 3.6
2014	SOI-GDL small	Swiss Ornithological Institute, Sempach, CH	0.64	Yes	46.2 ± 1.9	44.2 ± 2.6
2015	SOI-GDL2	Swiss Ornithological Institute, Sempach, CH	0.73	Yes	44.6 ± 3.4	39.6 ± 2.5
2016	SOI-GDL PAM	Swiss Ornithological Institute, Sempach, CH	1.72	Yes	43.6 ± 4.4	41.9 ± 6.2
2017	SOI-GDL3_ PAM	Swiss Ornithological Institute, Sempach, CH	1.60	No	40.9 ± 2.7	42.5 ± 2.8
2018	SOI-GDL PAM & nanoFix^TM^GEO-Mini	Swiss Ornithological Institute, Sempach, CH & PathTrack Ltd, Otley, UK	1.48 & 1.30	No	45.2 ± 3.6	40.5 ± 3.7
2019	nanoFix^TM^GEO-Mini	PathTrack Ltd, Otley, UK	1.30	No	44.8 ± 2.5	42.0 ± 4.7

#### Logger birds and non-logger birds

At the end of each breeding period in July/August, we picked birds for tagging which were in good condition, i.e. weighted at least 36 g and had a wing length of at least 169 mm. We did this to minimize the relative extra load by the logger for reasons of animal welfare. We tagged only birds, which had bred at least once successfully in our colony before because breeders are more faithful to the colony than non-breeders. Non-logger birds were those which had bred at least once successfully or showed at least a breeding attempt in the colony before but had never been tagged.

### Data sets and analyses

#### Return rate

We used antenna data (see above) of the logger birds and those of the same number of randomly chosen non-logger birds to detect returning birds in each year. We calculated the return rate as number of birds returned in year x + 1 / number of same birds returned in year x for each year between 2013 and 2020, and compared the rate of returned logger birds with the rate of returned non-logger birds using a generalized linear mixed model (GLMM) with binomial error distribution (number returned and not returned bound together as dependent variable) including a random slope for the logger-effect and random intercept grouped by study year. We also analysed whether the return rate of the logger birds (including repeatedly tagged individuals) was influenced by logger types with a light stalk (used 2014–2016), which might cause aerodynamic drag, and by sex coded as factor in a GLMM with binomial error distribution (returned “yes/no” as dependent variable). We used “logger type”, “year” and “bird’s ID” as random factors.

#### Apparent survival and recapture probability

Based on encounter data (i.e. capture history) of logger and non-logger birds from 2012 to 2020 (including repeated tagged individuals), we analysed apparent survival ϕ and recapture probability *p* in a capture-recapture analysis with Mark ver. 9.0 [44] using the R interface “RMark” [45]. We applied a Cormack-Jolly-Seber (CJS) model with “geo” (i.e. “tagged” or “not tagged”) and “sex” as factor variables to define groups and “year” as covariates. Based on corrected Akaike information criterion (AICc) we ranked models with these variables considering only models with a ΔAICc < 2 from the model with the lowest AICc (i.e. best model) [46].

#### Body weight, wing length and sex of logger birds and non-logger-birds

We measured body weight with an electric scale to the nearest 0.01 g (Kern und Sohn GmbH, Solingen, Germany), and wing length (maximum chord) [47] to the nearest 0.5 mm in July to August in each year, in case of logger birds (first tagging in repeatedly tagged birds) during tagging. We compared the body weight and wing length of logger birds with those of non-logger birds using linear mixed models (LMM) with “year” as random factor. Additionally, we checked for any size differences in body weight and wing length taken during tagging between logger birds that returned to the colony next year and those who did not return to the colony using a LMM with “year” as random factor. Since swifts are sexually monomorphic, we performed molecular sexing to determine sex [48].

#### Arrival date

We recorded arrival dates as the first registration of birds detected by the antenna system. Additionally, we fixed an iButton™ temperature logger (type DS1922L; accuracy ± 0.5 °C; Maxim Integrated™, USA) into the wall of each nest to measure nest temperature as a proxy for first use of the nest together with video surveillance using IR cameras (Conrad Electronics SE, Hirschau, Germany), and data from geolocators [49] to receive arrival dates. We investigated whether once or repeatedly tagging and/or wing length and/or body mass have an effect on arrival date using linear regression models (relation weight and wing length to arrival) and a LMM with “year” as random effect to compare arrival of logger with non-logger birds.

#### Breeding parameter

During each breeding season between April and August, we checked each nest every second day and tested whether logger birds differed from non-logger birds in time lag between arrival and clutch initiation (= “delta”), date of 1st egg (= “eggdate”), clutch size (= number of eggs laid), number of chicks, and number of fledglings. We also looked for sex-specific differences in all models using males and females which were no within-pair mates (with two exceptions) using LMMs (for delta and eggdate) or GLMMs with a Poisson error distribution (for clutch size, numbers of chicks and fledglings) with “year” and “nestID” as crossed random factors.

#### Arrival date and breeding parameter in logger birds tagged once on within-individual level

We checked whether there is an effect of tagging on individual level by comparing arrival date and breeding parameters in same individuals when they were tagged and when they were not tagged in different years. For this we used LMMs or GLMMs (with Poisson error distribution for counting data) with arrival date, date of clutch initiation, timeframe between arrival and clutch initiation, number of eggs, number of chicks, or number of fledglings as depending variable and “geo” (i.e. “tagged” or “not tagged”) as explanatory variable. “Bird’s ID” and “year” were used as random factors. According to Korner-Nievergelt [50], model assumptions for all LMMs and GLMMs were graphically assessed (e.g., normal distribution of residuals, QQ plots of residuals and random effects). Presence of overdispersion in GLMMs were checked with the function “dispersion_glmer” from the package “blmeco” [50].

#### Arrival date and laying date in repeatedly tagged logger birds on within-individual level

To check whether variance in arrival date and/or laying date in repeatedly tagged swifts differ between individuals from within-individuals, we conducted a repeatability analysis using data of repeatedly tagged birds. For this, we used an ANOVA-based method. We applied the function “rpt” from the R-package “rptR” for calculating confidence intervals and p-values [51, 52].

All statistical models were conducted with the software R (version 4.1.0) [53]. The level of significance was set to α = 0.05, and all average values are given as mean ± SD when not stated differently. Graphics were done with the R package “ggplot2” [54], “ggsignif” [55] and “ggpubr” [56].

## Results

### Return rate and logger type

In total, we tagged 85 birds within 8 years. Because we tagged some birds repeatedly, these 85 logger birds included 66 individual swifts. In total, 50 of 85 logger birds returned to the colony during the period between 2013 and 2020. On average logger birds returned with a rate of 0.61 ± 0.12 and non-logger birds returned with a rate of 0.60 ± 0.27 to the breeding site in the next year (Table 2, GLMM, estimate_logger_ = − 0.159 ± 0.600, N_years of logger birds_ = M_years of non-logger birds_ = 8, z = − 0.265, *P* = 0.791). Looking only at birds that have been tagged the first time, 39 of 66 logger birds returned (0.59 ± 0.23, Table 2).

**Table 2:** Total number of breeding pairs per year and number and rate of returned logger and non-logger birds for each year

Year (x)	Logger-birds						Non-logger birds		
	Total N oof breeding pairs	No. of all birds tracked in year x	No. of all tracked birds returned in year (x + 1)	No. of birds tracked for the first time in year x	No. of first tracked birds returned in year (x + 1)	Return rate	No. of birds sampled for comparison in year x	No. of same birds returned in year (x + 1)	Return rate
2012	44	10	8	10	8	0.8	10	3	0.3
2013	42	10	7	6	5	0.7	10	6	0.6
2014	48	10	6	6	4	0.6	10	4	0.4
2015	47	10	5	8	4	0.5	10	9	0.9
2016	53	10	7	5	5	0.7	10	7	0.7
2017	58	11	6	9	4	0.55	11	9	0.82
2018	59	19	8	19	8	0.42	19	17	0.89
2019	60	5	3	3	1	0.6	5	1	0.2
Sum		85	50	66	39	0.61	85	56	0.6

Out of the 66 logger-birds tagged first time, 16 individuals carried a logger at least for two years (12 returned in the third year ≙ 0.75). Seven of these 16 individuals were tagged in three years (5 returned in the fourth year ≙ 0.71), four individuals were tagged four times (2 returned in the fifth year ≙ 0.5) and one female was tagged five times but did not return in the sixth year.

There was neither an effect of the presence of a light stalk nor of sex on the return rate of logger birds including repeated tagging (LMM, estimate_stalk_ = 0.136 ± 0.503, N_logger bird incl. repeated tagging_ = 94, z = 0.270, *P* = 0.787; estimate_female_ = 0.613 ± 0.490, N_sexed logger bird incl. repeated tagging_ = 92, z = 1.251, *P* = 0.211).

#### Apparent survival ϕ and recapture probability p

We found three CJS models with a similar AICc as the best model (ΔAICc < 2), but none of these four models included the factor “geo” (i.e. “tagged” or “not tagged”) as grouping variable for survival ϕ. The factor “geo” together with “sex” was included by model selection for the recapture probability (model 1: ϕ(~ 1) + *p*(~ geo + sex), AICc = 582.23; model 2: ϕ(~ year) + *p*(~ geo + sex), AICc = 582.87; model 3: ϕ(~ 1) + *p*(~ geo), AICc = 583.05; model 4: ϕ(~ sex) + *p*(~ geo + sex), AICc = 584.15). Survival estimated by the best model 1 was 0.727 ± 0.021 for all birds; when considering the covariate “year” in model 2, ϕ was 0.731 ± 0.021, and when considering “sex” as grouping factor in model 4, ϕ of females (0.719 ± 0.032) was about 2% lower than ϕ of males (0.734 ± 0.028). Recapture probability *p* was 1.0 ± 0.0 for untagged birds in all four models. For tagged birds, *p* was 0.948 ± 0.021 for both sexes in model 3 and stated 0.982 ± 0.018 for males in models 1, 2 and 4, and varied between 0.914 ± 0.036 (model 2), 0.916 ± 0.036 (model 1) and 0.917 ± 0.035 (model 4) for females, respectively.

#### Body weight and wing length of logger birds and non-logger-birds

**Fig. 1:**
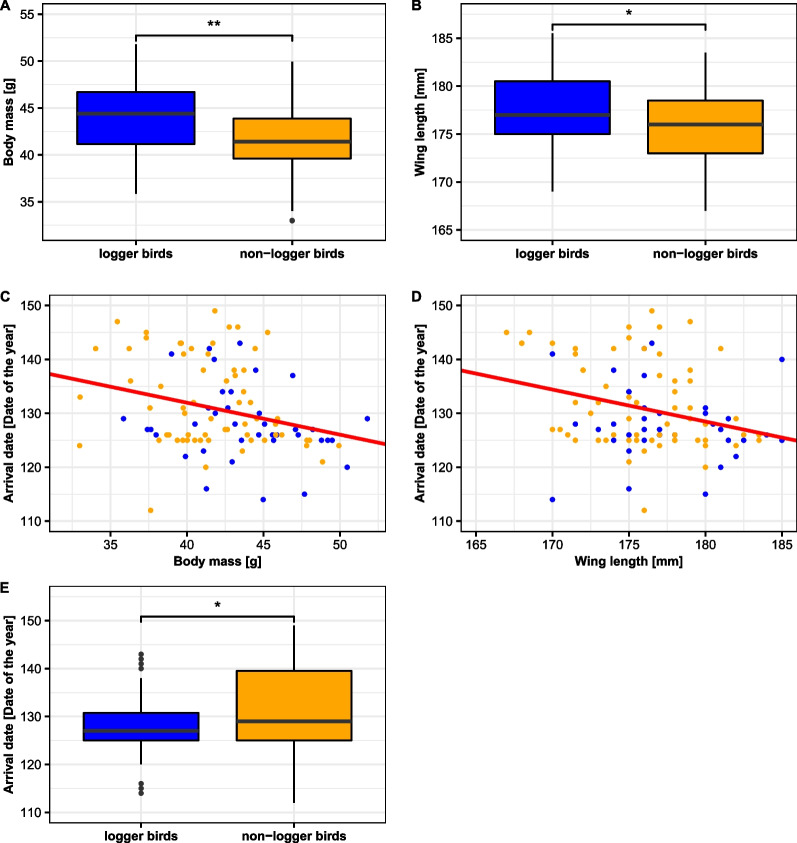
Body weight [g] (**A**) and wing length [mm] (**B**) of logger birds (blue) and non-logger birds (orange). Regression between body weight and arrival date (**C**) and between wing length and arrival date (**D**) in logger and non-logger birds. Arrival date of logger and non-logger birds (**E**). Black dots are outliers, i.e. values that are less or greater than 1.5 times the interquartile range

We received data on body weight and wing length from 66 logger birds (31 females, 33 males, 2 unknown) during tagging in year x and from 63 non-logger birds (28 females, 34 males, 1 sex unknown). Logger birds were significantly heavier (LMM, estimate_logger_ = 2.173 ± 0.652, N_logger birds_ = 66, M_non-logger birds_ = 63, df = 128.8, t = 3.334, *P* = 0.001) and significantly larger in wing length (LMM, estimate_logger_ = 1.638 ± 0.697, N_logger birds_ = 66, M_non-logger birds_ = 63, df = 128.0, t = 2.351, *P* = 0.020) than non-logger birds (Fig. 1A, B) in the year of logger deployment. We could not detect any significant differences in body weight and wing length between returned logger birds and those logger birds which did not return (LMM, weight: estimate_returned_ 0.414 ± 0.844, N_returnees_ = 37, M_non-returnees_ = 29, df = 63.4, t = 0.491, *P* = 0.625; wing: estimate_returned_ 0.064 ± 0.998, N_returnees_ = 37, M_non-returnees_ = 29, df = 65.0, t = 0.064, *P* = 0.949). Although we set a minimum weight and wing length for logger birds, we still covered a wide range in weight and size in the species (logger birds: range in weight: 35.9–51.8 g; range in wing length: 169.0–185.5 mm).

#### Arrival date

We received arrival dates of 101 individuals (50 females, 50 males, 1 unknown, including N = 38 logger and N = 63 non-logger birds) and detected a negative relation between body weight and arrival date (arrival = 155.7 − 0.6*weight, N = 101, R^2^_adjusted_ = 0.073, *P* = 0.004) and between wing length and arrival date (arrival = 235.3 − 0.6*wing, N = 101, R^2^_adjusted_ = 0.077, *P* = 0.003; Fig. 1C, D). Logger birds arrived earlier at the breeding site than non-logger birds (LMM, estimate_logger_ = − 3.620 ± 1.382, N_logger- birds_ = 38, M_non-logger- birds_ = 63, df = 95.8, t = − 2.619, *P* = 0.010; Fig. 1E), following the general pattern that heavier and larger birds arrived earlier at the breeding site than lighter and smaller birds.

#### Breeding parameter

**Fig. 2:**
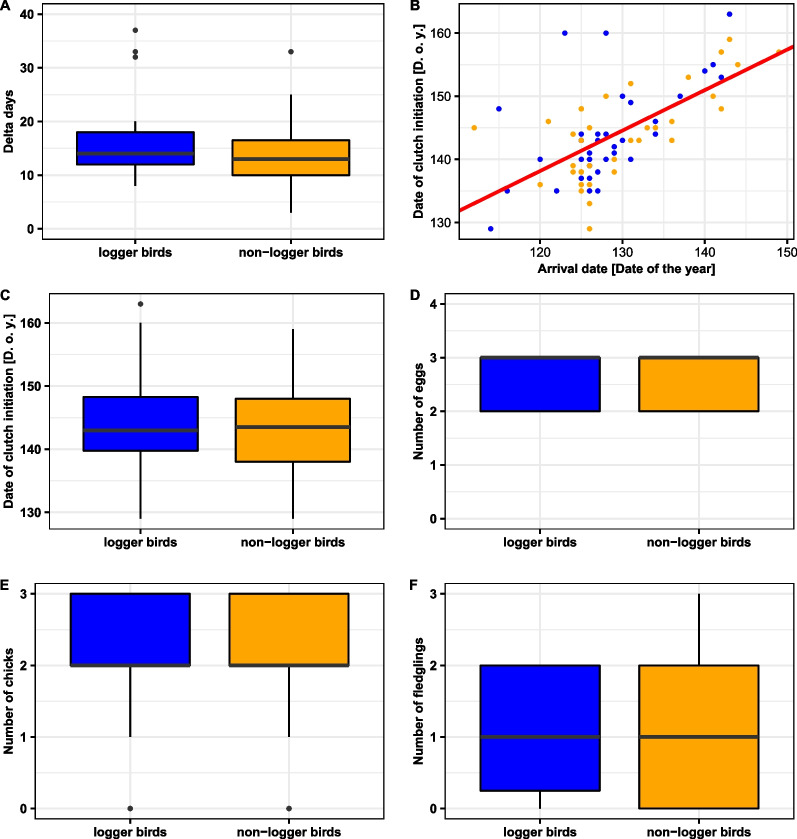
Timeframe between arrival and clutch initiation [delta days] (**A**) of logger birds (blue) and non-logger birds (orange), (**B**) relation between arrival date and laying first egg, (**C**) date of clutch initiation [Day of the year], (**D**) clutch size, (**E**) number of chicks, (**F**) number of fledglings in logger birds and non-logger birds. Black dots are outliers, i.e. values that are less or greater than 1.5 times the interquartile range

The timeframe between arrival and starting egg laying (i.e. delta days) did not differ between logger and non-logger birds (LMM, estimate_logger_ = 2.089 ± 1.319, N_logger birds_ = 36, M_non-logger birds_ = 36, df = 55.3, t = 1.583, *P* = 0.119, Fig. 2A). When combining data of logger and non-logger birds, we found a strong positive relation between arrival date and the laying date of the first egg (eggdate = 61.3 + 0.6*arrival, N = 72, R^2^_adjusted_ = 0.396, *P* < 0.001, Fig. 2B). We received data on breeding parameters recorded between 2013 and 2020 in 36 logger birds (21 females, 15 males) and 38 non-logger birds (14 females, 24 males). We did not detect any differences in breeding parameters between both groups. They started egg laying at the same time (LMM, estimate_logger_ = 0.172 ± 1.371, N_logger bird_ = 36, M_non-logger birds_ = 36, df = 61.2, t = 0.125, *P* = 0.901, Fig. 2C), had similar clutch sizes (GLMM, estimate_logger_ = − 0.043 ± 0.147, N_logger birds_ = 36, M_non-logger birds_ = 38, z = − 0.293, *P* = 0.769, Fig. 2D), a similar number of chicks (GLMM, estimate_logger_ = − 0.026 ± 0.165, N_logger birds_ = 34, M_non-logger birds_ = 38, z = − 0.160, *P* = 0.873, Fig. 2E), and a similar number of fledglings (GLMM, estimate_logger_ = 0.107 ± 0.227, N_logger birds_ = 34, M_non-logger birds_ = 38, z = 0.469, *P* = 0.639, Fig. 2F). Females and males, or rather their female mates (both logger and non-logger, no within-pair mates, with two exceptions) did not differ in date of clutch initiation (LMM, estimate_female_ = 0.372 ± 1.368, N = 33 females, M = 39 males, df = 57.7, t = 0.272, *P* = 0.787), clutch size (GLMM, estimate_female_ = -0.039 ± 0.145, N = 35 females, M = 39 males, z = − 0.271, *P* = 0.786), number of chicks (GLMM, estimate_female_ = -0.129 ± 0.165, N = 34 females, M = 38 males, z = − 0.785, *P* = 0.433) and number of fledglings (GLMM, estimate_female_ = − 0.086 ± 0.226, N = 34 females, M = 38 males, z = − 0.382, *P* = 0.703).

#### Arrival date and breeding parameter in same birds when tagged and when not tagged

From the 16 repeatedly tracked swifts, we received breeding data of 15 of these same individuals when they were tagged and when they were not tagged for at least one year. None of the LMMs could detect any significant effect of loggers on any of the factors mentioned (Table 3).

**Table 3:** Results from LMMs (“arrival”, “eggdate” and “delta”) and from GLMM (“clutch”, “chicks”, “fledge”) testing for associations between having a logger (“geo”, df = 1) and arrival date (“arrival”), date of clutch initiation (“eggdate”), timeframe between arrival and clutch initiation (“delta”), number of eggs (“clutch”), number of chicks (“chicks”) and number of fledglings (“fledge”)

Formula	N	Estimate (± SE)	t/z-value	*P*-value
arrival ~ geo + (1 | ID) + (1 | Year)	15	1.657 (± 1.315)	1.260	0.215
eggdate ~ geo + (1 | ID) + (1 | Year)	15	0.359 (± 1.450)	0.248	0.805
delta ~ geo + (1 | ID) + (1 | Year)	15	− 0.509 (± 1.822)	− 0.279	0.781
clutch ~ geo + (1 | ID) + (1 | Year)	15	− 0.028 (± 0.165)	− 0.169	0.866
chicks ~ geo + (1 | ID) + (1 | Year)	15	0.120 (± 0.186)	0.645	0.521
fledge ~ geo + (1 | ID) + (1 | Year)	15	− 0.064 (± 0.220)	− 0.292	0.771

We detected no significant differences in arrival date, egg laying date, time span between arrival and laying of first egg, clutch size, number of chicks and number of fledglings in these birds when they were tagged or were not tagged.

#### Arrival date and laying date in repeatedly tagged logger birds on within-individual level

Out of 66 logger-birds, 16 swifts were tagged at least for two years. Of these 16 common swifts, we got 31 arrival dates of the 12 tagged and returned swifts and 30 dates of clutch initiation (“eggdate”) of 11 individuals for repeatability analysis. We detected a high within-individual consistency in arrival date (arrival: N = 12, R (± SE): 0.6 ± 0.173, *P* < 0.001) and moderate repeatable timing in egg laying (egg date: N = 11, R (± SE): 0.34 ± 0.201, *P* = 0.040).

## Discussion

Using a long-term data set of 66 common swifts tagged once or repeatedly and 63 non-logger birds from the same breeding colony in Germany, we detected no differences between logger and non-logger birds in different traits regarding apparent survival and life-history traits over eight years. The return rate of once tagged and repeatedly tagged logger birds did not differ from the return rate of non-logger birds, and the return rates were similar to return rates of other bird species of similar body weight or even less [17, 20, 21]. According to the capture-recapture analysis, apparent survival was similar for logger and non-logger birds and for both sexes. As the recapture probability where high (> 0.9) for logger and non-logger birds, we can assume that the determined survival of rounded 0.73 is a fairly accurate estimate for the “true” survival [57]. We received a high recapture probability because the antenna system allows an increased encounter rate of the RFID-tagged birds. In contrast to this study, Morganti et al. [25] found an effect on apparent survival in 11 different swift colonies located in Italy, Spain, or Sweden. Swifts carrying a geolocator had on average a 26.69% lower apparent survival than non-logger birds. They also detected a significant heterogeneity in return rates among sites, possibly due to site-specific recapture probabilities, which they could not control for. The method of capture might lower the apparent survival in both logger birds and non-logger birds because the number of birds that returned and were recognized, is crucial to calculate the apparent survival. In our study we compared logger-birds with non-logger birds from the same colony. Thus, we had no “colony effect”. Second, Morganti et al. [25] captured adults at their nest or adults were captured with mist nests from outside the building in front of the entrance of the nests. In our study we used our antenna system which automatically reported the presence of an individual bird when it enters the nest. Thus, with our system, we do not need to capture a bird to get the identity of that bird. That might be a reason, why our return rate is higher in logger and non-logger birds than the return rate in the colonies studied by Morganti et al.

Logger birds arrived earlier than non-logger birds at the breeding site in spring, following the general pattern with larger swifts and those heavier in body weight arriving earlier at the breeding site [16]. The timeframe between arrival and clutch initiation did not differ between logger and non-logger birds. Thus, logger birds fitted to the pattern that “early birds” started egg laying earlier (Fig. 2B). This is important, because timing of breeding is crucial for the reproductive success of a complete breeding season [58, 59]. The fact, that logger birds did not delay clutch initiation is also important in respect to another aspect. Due to technical reasons, it was necessary to recapture the logger bird between arrival and clutch initiation to retrieve the logger to download the data. Catching a bird during this time, however, might have been a major negative impact on the breeding success due to a delayed clutch initiation [60, 61] or even nest desertion in this sensitive bird, but this was not the case in our study.

Our analysis showed that logger birds, regardless of sex, were as successful in reproduction as non-logger birds in our breeding colony. We found no differences in date of clutch initiation, in number of eggs, number of chicks nor in number of fledglings. Thus, we detected no “carry-over” effects neither on a between-individual level nor on a within-individual level, i.e. investing the same individuals when they were tagged during the previous non-breeding season vs. when they were not tagged. Due to reasons for animal welfare, we set the minimum weight and wing length for logger birds. In comparison to the natural range in weight and wing length, we still covered almost the natural range in these traits in our logger birds (wing: 166–187 mm in males and 163–183 mm in females and weight: 31–56 g) [62]. To be on a safe side, we recommend ornithologist to logger only birds in good shape and a good weight to avoid negative impacts of tagging devices especially in small bird species. Our repeatability analysis showed that logger birds were highly consistent in arrival date and moderately consistent in date of clutch initiation. We detected this individual consistency in arrival date in another data set of repeatedly observed 26 males and 28 females of our long-term study of this breeding colony as well (unpublished data). Thus, logger birds exhibit similar patterns like non-logger birds. Further, high consistency in arrival at the breeding site was also found in other studies with migratory bird species [32, 34]. Therefore, we assume that common swifts have their individual timing for arriving at the breeding site and starting egg laying following their internal clock [63]. It would be interesting to check, whether the timing of arrival and egg laying exist already with the first breeding attempt or will be developed during years of breeding experience [64]. A study by Sergio et al. [36] investigated the performance in migration in black kites (*Milvus migrans*) during life and compared young migrants with migrants of middle age and old kites. The migratory performance was mediated by within-individual improvements and selective mortality. Kites performed gradually better with getting older. Early life stages seem to be an important phase for migration performance later on in life.

Since we have indications that common swifts do have their own timing of life [repeatability analysis, 13], we suggest that future studies should look more into traits on within-individual level rather than into between-individual differences. In another study on common swifts of this breeding colony, we could receive migration routes and overwintering sites of three males over two successive overwintering periods [13]. We found that all three males used different migration routes and overwintering areas, but each male used the same routes and regions in two successive wintering periods. This emphasizes the difference between individuals and the consistency within individuals in one trait in this species.

Although we found no negative effect of tagging once or repeatedly in swifts, tagging remains an important issue. The current loggers for small birds do not allow real-time monitoring. Thus, we can only examine logger birds that actually returned to the breeding site, but we have no information about the “non-returnees” and could only speculate whether they are dead or breed at another breeding site. It is possible that negative effects of tagging are masked by the fact that the returnees were in the better physical condition and could compensate for possible negative effects during non-breeding period and arrived at the breeding site. However, we found no difference in the body weight nor in wing length between returnees and non-returnees when they were tagged in year x.

Another study on common swifts and pallid swifts *Apus pallidus* revealed a reduced apparent survival on logger birds comparing to non-logger birds as their control group [25]. It seems that the weight of the logger did not influence the survival but the logger characteristics. When the logger was equipped with a light stalk, the apparent survival was lower indicating that the logger set up might have a major impact on the return rate [25]. Although our sample size was small, we took a look at the type of the logger, i.e. with or without a light stalk. We did not find any effects combined with sex as fixed effect on the return rate in logger birds. Nevertheless, the study [25] makes a significant point that it could be far more important how a logger is built and shaped rather than just focusing on weight, as aerodynamics matters a lot in birds, especially in long-distance migratory species [65].

There is still an ongoing debate about possible negative effects of tagging in birds [66]. Some studies detected negative effects especially in small bird species [67]. Small logger birds can have a lower return rate [68], or when returned, reproduction is delayed and clutches are smaller [69]. In lesser kestrels *Falco naumanni* tagged pairs had greater fledgling mortality in the following breeding season [70]. Thus, we should continue to investigate effects of tagging on a variety of traits, including even effects on young of tagged parents [70].

To better understand individual based decisions regarding life-history traits, we need more studies using repeatedly tracked birds [13, 14]. Although we found no differences in return rate and parameters determine breeding success in repeatedly tagged swifts in our breeding colony, further long-term studies are essential to evaluate effects of such repeatedly used techniques to get more knowledge on possible impacts on migration behaviour and reproductive success in long-distance migratory birds in general.

## Conclusion

Our study confirms that common swift tagged once or repeatedly with different types of loggers has no effect on apparent survival and breeding performance in comparison to non-logger birds. Even on within-individual level, we found no difference in any breeding parameter showing that the individual behaviour did not change due to tagging once or repeatedly. Nonetheless, we encourage other scientists working with tracking devices in birds to check for any “carry-over” effects due to logging on a bird’s life.

## Data Availability

All data analysed during the current study are available from the corresponding author upon reasonable request.

## Abbreviations

AICc: Corrected Akaike information criterion
CJS: Cormack-Jolly-Seber
LMM: Linear mixed effects model
GLMM: Generalized linear mixed model
RFID: Radio-frequency identification

## References

[1] Gordo O, Brotons L, Ferrer X, Comas P (2005). Do changes in climate patterns in wintering areas affect the timing of the spring arrival of trans-Saharan migrant birds?. Glob Chang Biol.

[2] Both C, Visser ME (2001). Adjustment to climate change is constrained by arrival date in a long-distance migrant bird. Nature.

[3] Alerstam T (1993). Bird migration.

[4] Alerstam T, Hedenström A, Åkesson S (2003). Long-distance migration: evolution and determinants. Oikos.

[5] Harrison AX, Blount JD, Inger R, Norris DR, Bearhop S (2010). Carry-over effects as drivers of fitness differences in animals. J Ecol.

[6] Crysler ZJ, Ronconi RA, Taylor PD (2016). Differential fall migratory routes of adult and juvenile Ipswich Sparrows (*Passerculus sandwichensis princeps*). Mov Ecol.

[7] Mancuso KA, Fylling MA, Bishop CA, Hodges KE, Lancaster MB, Stone KR (2021). Migration ecology of western gray catbirds. Mov Ecol.

[8] Schmaljohann H (2019). The start of migration correlates with arrival timing, and the total speed of migration increases with migration distance in migratory songbirds: a cross-continental analysis. Mov Ecol.

[9] Ouwehand J, Ahola MP, Ausems ANMA, Bridge ES, Burgess M, Hahn S (2016). Light-level geolocators reveal migratory connectivity in European populations of pied flycatchers *Ficedula hypoleuca*. J Avian Biol.

[10] Stutchbury BJM, Tarof SA, Done T, Gow E, Kramer PM, Tautin J (2009). Tracking long-distance songbird migration by using geolocators. Science.

[11] Briedis M, Bauer S, Adamík P, Alves JA, Costa JS, Emmenegger T (2019). A full annual perspective on sex-biased migration timing in long-distance migratory birds. Proc R Soc B.

[12] Klvaňa P, Cepák J, Munclinger P, Michálková R, Tomášek O, Albrecht T (2018). Around the Mediterranean: an extreme example of loop migration in a long-distance migratory passerine. J Avian Biol.

[13] Wellbrock AHJ, Bauch C, Rozman J, Witte K (2017). ‘Same procedure as last year?‘ Repeatedly tracked swifts show individual consistency in migration pattern in successive years. J Avian Biol.

[14] McKinnon EA, Love OP (2018). Ten years tracking the migrations of small landbirds: lessons learned in the golden age of bio-logging. Auk.

[15] Geen GR, Robinson RA, Baillie SR (2019). Effects of tracking devices on individual birds - a review of the evidence. J Avian Biol.

[16] Åkesson S, Atkinson PW, Bermejo A, de la Puente J, Ferri M, Hewson CM (2020). Evolution of chain migration in an aerial insectivorous bird, the common swift *Apus apus*. Evolution.

[17] Delancey CD, Islam K, Kramer GR, MacDonald GJ, Sharp AR, Connare BM (2020). Geolocators reveal migration routes, stopover sites, and nonbreeding dispersion in a population of Cerulean Warblers. Anim Migr.

[18] Verhoeven MA, Loonstra AHJ, McBride AD, Macias P, Kaspersma W, Hooijmeijer JCEW (2020). Geolocators lead to better measures of timing and renesting in black-tailed godwits and reveal the bias of traditional observational methods. J Avian Biol.

[19] Norris DR, Marra PP (2007). Seasonal interactions, habitat quality, and population dynamics in migratory birds. Condor.

[20] Bell SC, El Harouchi M, Hewson CM, Burgess MD (2017). No short- or long-term effects of geolocator attachment detected in Pied Flycatchers *Ficedula hypoleuca*. Ibis.

[21] Mondain-Monval TO, Du Feu R, Sharp SP (2020). The effects of geolocators on return rates, condition, and breeding success in common Sandpipers *Actitis hypoleucos*. Bird Study.

[22] Pakanen VM, Rönkä N, Thomson RL, Koivula K (2015). No strong effects of leg-flagged geolocators on return rates or reproduction of a small long-distance migratory shorebird. Ornis Fenn.

[23] Kürten N, Vedder O, González-Solís J, Schmaljohann H, Bouwhuis S (2019). No detectable effect of light-level geolocators on the behaviour and fitness of a long-distance migratory seabird. J Ornithol.

[24] van Wijk RE, Souchay G, Jenni-Eiermann S, Bauer S, Schaub M (2016). No detectable effects of lightweight geolocators on a Palaearctic-African long-distance migrant. J Ornithol.

[25] Morganti M, Rubolini D, Åkesson S, Bermejo A, de la Puente J, Lardelli R (2018). Effect of light-level geolocators on apparent survival of two highly aerial swift species. J Avian Biol.

[26] Pakanen VM, Rönkä N, Thomson RL, Blomqvist D, Koivula K (2020). Survival probability in a small shorebird decreases with the time an individual carries a tracking device. J Avian Biol.

[27] Arlt D, Low M, Pärt T (2013). Effect of geolocators on migration and subsequent breeding performance of a long-distance passerine migrant. PLoS ONE.

[28] Scandolara C, Rubolini D, Ambrosini R, Caprioli M, Hahn S, Liechti F (2014). Impact of miniaturized geolocators on barn swallow *Hirundo rustica* fitness traits. J Avian Biol.

[29] Seward A, Taylor RC, Perrow MR, Berridge RJ, Bowgen KM, Dodd S (2021). Effect of GPS tagging on behaviour and marine distribution of breeding Arctic Terns *Sterna paradisaea*. Ibis.

[30] Weiser EL, Lanctot RB, Brown SC, Alves JA, Battley PF, Bentzen R (2016). Effects of geolocators on hatching success, return rates, breeding movements, and change in body mass in 16 species of Arctic-breeding shorebirds. Mov Ecol.

[31] Brlík V, Koleček J, Burgess M, Hahn S, Humple D, Krist M (2020). Weak effects of geolocators on small birds: a meta-analysis controlled for phylogeny and publication bias. J Anim Ecol.

[32] Stanley CQ, MacPherson M, Fraser KC, McKinnon EA, Stutchbury BJM (2012). Repeat tracking of individual songbirds reveals consistent migration timing but flexibility in route. PLoS ONE.

[33] Evens R, Beenaerts N, Witters N, Artois T (2017). Repeated migration of a juvenile European Nightjar Caprimulgus europaeus. J Ornithol.

[34] Kürten N, Schmaljohann H, Bichet C, Haest B, Vedder O, González-Solis J (2022). High individual repeatability of the migratory behaviour of a long-distance migratory seabird. Mov Ecol.

[35] López-López P, García-Ripollés C, Urios V (2014). Individual repeatability in timing and spatial flexibility of migration routes of trans-Saharan migratory raptors. Curr Zool.

[36] Sergio F, Tanferna A, De Stephanis R, López Jiménez L, Blas J, Tavecchia G, Preatoni D, Hiraldo F (2014). Individual improvements and selective mortality shape lifelong migratory performance. Nature.

[37] Pedersen L, Jackson K, Thorup K, Tøttrup AP (2018). Full-year tracking suggests endogenous control of migration timing in a long-distance migratory songbird. Behav Ecol Sociobiol.

[38] Lack D (1956). Swifts in a tower.

[39] Weitnauer E (1980). "Mein Vogel" Aus dem Leben des Mauerseglers Apus apus.

[40] Hedenström A, Norevik G, Warfvinge K, Andersson A, Bäckman J, Åkesson S (2016). Annual 10-month aerial life phase in the common swift Apus apus. Curr Biol.

[41] Åkesson S, Klaassen R, Holmgren J, Fox JW, Hedenström A (2012). Migration routes and strategies in a highly aerial migrant, the common swift *Apus apus*, revealed by light-level geolocators. PLoS ONE.

[42] Åkesson S, Bianco G, Hedenström A (2016). Negotiating an ecological barrier: crossing the Sahara in relation to winds by common swifts. Phil Trans R Soc B.

[43] Walker MD, Rozman J, Witte K (2009). Breeding colony of Common Swifts (*Apus apus*) in a motorway bridge. Vogelwarte.

[44] White GC, Burnham KP (1999). Program MARK: survival estimation from populations of marked animals. Bird Study.

[45] Laake JL. RMark: an R interface for analysis of capture–recapture data with MARK. Seattle: Alaska Fish. Sci. Cent. Processed Rep.; 2013.

[46] Symonds MR, Moussalli A (2011). A brief guide to model selection, multimodel inference and model averaging in behavioural ecology using Akaike’s information criterion. Behav Ecol Sociobiol.

[47] Svensson L (1992). Identification guide to European passerines.

[48] Wellbrock AHJ, Bauch C, Rozman J, Witte K (2012). Buccal swabs as a reliable source of DNA for sexing young and adult Common Swifts (Apus apus). J Ornithol.

[49] Schaub T, Wellbrock AHJ, Rozman J, Witte K (2019). Light data from geolocation reveal patterns of nest visit frequency and suitable conditions for efficient nest site monitoring in common swifts *Apus apus*. Bird Study.

[50] Korner-Nievergelt F, Roth T, von Felten S, Guelat J, Almasi B, Korner-Nievergelt P. Bayesian data analysis in ecology using linear models with R, BUGS and STAN. New York: Academic Press; 2015.

[51] Nakagawa S, Schielzeth H (2010). Repeatability for Gaussian and non-Gaussian data: a practical guide for biologists. Biol Rev.

[52] Stoffel MA, Nakagawa S, Schielzeth H (2017). rptR: repeatability estimation and variance decomposition by generalized linear mixed-effects models. Methods Ecol Evol.

[53] R Core Team. R: A language and environment for statistical computing*.* Vienna: R Foundation for Statistical Computing; 2021. https://www.R-project.org/. Accessed 04 Apr 2022.

[54] Wickham H (2016). ggplot2: elegant graphics for data analysis.

[55] Ahlmann-Eltze C, Patil I. ggsignif: R package for displaying significance brackets for 'ggplot2'. 2021. https://psyarxiv.com/7awm6/. Accessed 04 Apr 2022.

[56] Kassambara A. ggpubr: 'ggplot2' based publication ready plots. 2020. https://CRAN.R-project.org/package=ggpubr. Accessed 04 Apr 2022.

[57] Cooch EG, White GC. Program MARK. A gentle introduction. 2021. http://www.phidot.org/software/mark/docs/book/. Accessed 27 Oct 2022.

[58] McKellar AE, Marra PP, Ratcliffe LM (2013). Starting over: experimental effects of breeding delay on reproductive success in early-arriving male American redstarts. J Avian Biol.

[59] Velmala W, Helle S, Ahola MP, Klaassen M, Lehikoinen E, Rainio K (2015). Natural selection for earlier male arrival to breeding grounds through direct and indirect effects in a migratory songbird. Ecol Evol.

[60] Low M, Arlt D, Pärt T, Öberg M (2015). Delayed timing of breeding as a cost of reproduction. J Avian Biol.

[61] Öberg M, Pärt T, Arlt D, Laugen AT, Low M (2014). Decomposing the seasonal fitness decline. Oecologia.

[62] Demongin L. Identification guide to birds in the hand. 2016. Beauregrad-Vendon, L. Demongin (published privately); 2016 France.

[63] Åkesson S, Ilieva M, Karagicheva J, Rakhimberdiev E, Tomotani B, Helm B (2017). Timing avian long-distance migration: from internal clock mechanisms to global flights. Phil Trans R Soc B.

[64] Wimerskirch H (1992). Reproductive effort in long-lived birds: age-specific patterns of condition, reproduction and survival in the wandering albatross. Oikos.

[65] Bowlin MS, Henningsson P, Muijres FT, Vleugels RHE, Liechti F, Hedenström A (2010). The effects of geolocator drag and weight on the flight ranges of small migrants. Methods Ecol Evol.

[66] Constantini D, Moller AP (2013). A meta-analysis of the effects of geolocator applications on birds. Curr Zool.

[67] Fairhurst GD, Berzins LL, Bradley DW (2015). Assessing costs of carrying geolocators using feather corticosterone in two species of aerial insectivore. R Soc Open Sci.

[68] Taff CC, Freeman-Gallant CR, Streby HM, Kramer GR (2018). Geolocator deployment reduces return rate, alters selection, and impacts demography in a small songbird. PLoS ONE.

[69] Scandolara C, Ambrosini DR, Caprioli M (2014). Impact of miniaturized geolocators on barn swallow Hirundo rustica fitness traits. J Avian Biol.

[70] Rodríguez A, Negro JJ, Fox JW, Afanasyev V (2009). Effects of geolocator attachments on breeding parameters of Lesser Kestrels. J Field Ornithol.

